# Decoding non-human mammalian adaptive signatures of 2.3.4.4b H5N1 to assess its human adaptive potential

**DOI:** 10.1128/spectrum.00948-25

**Published:** 2025-08-11

**Authors:** Ranjana Nataraj, Avinash Karkada Ashok, Ayushi Amin Dey, Sannula Kesavardhana

**Affiliations:** 1 Department of Biochemistry, Division of Biological Sciences, Indian Institute of Science 29120, Bengaluru, Karnataka, India; Regional Centre for Biotechnology, Faridabad, Haryana, India

**Keywords:** HPAIs, avian influenza, 2.3.4.4b H5N1, human adaptation, non-human mammals, virus evolution, host-directed evolution, panzootic virus, hemagglutinin, virus polymerase complex

## Abstract

The 2.3.4.4b clade highly pathogenic avian influenza H5N1 infected diverse non-human mammalian species, gained mammal-to-mammal transmission potential, and caused sporadic human infections. However, whether non-human mammals enable the human adaptation of 2.3.4.4b H5N1 to establish human infections is unclear. Gain-of-function research restrictions may hinder the assessment of 2.3.4.4b H5N1 human adaptations. Here, we tracked the evolution of 2.3.4.4b H5N1 that infected non-human mammals and evaluated their ability to gain human adaptations. The non-human mammal 2.3.4.4b H5N1 partly acquired classical human-adapting mutations, which are identical to the residues of H1N1pdm09 and seasonal human H3N2 viruses, while showing a few species-specific adaptations that might be potential barriers for successful human infections. The polymerase complex proteins, PA and PB2, acquired human adaptations in non-human mammals, with fox-infected viruses showing more positive selection in the polymerase complex. The human-adapting Q591K/R substitution in PB2 appeared only in the 2.3.4.4b clade but not in previously circulating H5N1 strains. Despite minimal changes in hemagglutinin (HA), A160T and T199I mutations near the receptor binding site of HA in dairy cattle viruses indicate the rapid HA glycan surface evolution affecting virus entry and immune evasion. The unbiased quantitative assessment of virus adaptations indicated that 2.3.4.4b H5N1 circulating in bears, cattle, dolphins, and foxes might show better human adaptive potential. Thus, 2.3.4.4b H5N1 appears to be acquiring human adaptations due to natural selection pressure in non-human mammals. Overall, our study delineates human adaptation and infection risk of specific non-human mammalian circulating 2.3.4.4b H5N1 strains.

**IMPORTANCE:**

The 2.3.4.4b clade H5N1 virus emerged as a panzootic strain, leading to the unprecedented deaths of domestic and wild birds and diverse non-human mammalian species. Intriguingly, the 2.3.4.4b H5N1 transmitted to diverse mammalian species and gained mammal-to-mammal transmission, suggesting its pandemic potential. The H5N1 outbreaks in dairy cattle and sea lions are devastating, and they contributed to sporadic human infections. This indicates the ability of non-human mammal hosts, like dairy cattle, as potential sources for human transmission. However, the signatures of non-human mammal adaptations of 2.3.4.4b H5N1 and how these adaptations drive the human adaptive potential of 2.3.4.4b H5N1 are unclear. In this study, we show the specific molecular patterns of H5N1 proteins that determine species-specific adaptations in non-human mammals. We identified that 2.3.4.4b H5N1 circulating in non-human mammals is rapidly evolving with critical adaptations in PA, PB2, and HA and gaining human adaptive potential in specific non-human mammalian species.

## INTRODUCTION

Highly pathogenic avian influenza (HPAI) viruses circulate in poultry and domestic birds and often reassort with wild bird influenza subtypes. In particular, the Goose/Guangdong (Gs/GD) lineage HPAI H5N1 causes frequent outbreaks in poultry and domestic birds and is occasionally transmitted to humans. The H5N1 is the common strain that has dominated circulating avian influenza viruses from the Gs/GD lineage. The recent 2.3.4.4 clade Gs/GD H5 viruses reassorted with diverse neuraminidase (NA) subtypes (H5N2, H5N5, H5N6, and H5N8) and appeared in circulating avian influenza viruses and caused human infections. However, the H5N1 subtype resurged due to 2.3.4.4b H5 pairing with N1 and showed unprecedented infections and spread in diverse species (1–4). This virus acquired the ability to spread through wild birds with migratory behavior, which enabled transmission to various marine and terrestrial mammalian species globally and reassorted with geographically diverse low pathogenic avian influenza viruses (5–10). H5N1 is adapted to replicate primarily in avian species. Its ability to replicate and spread in mammals requires specific adaptations in its glycoproteins, hemagglutinin (HA), NA, and polymerase complex required for virus replication and transcription. Recent studies demonstrate that 2.3.4.4b H5N1 viruses were able to spread in infected cattle (11), minks (3), sea lions (2), and experimentally infected ferrets (11). This may enable the emergence of novel strains suitable to replicate and propagate in mammals, including humans, thus posing a threat to public health and the economy.

The sudden surge in recent human H5N1 infections suggests the potential risk of human outbreaks if the 2.3.4.4b H5N1 gains adaptations to replicate efficiently in human cells and show human-to-human transmission (12, 13). The 2.3.4.4b H5N1 spread to diverse non-human mammalian species, like cattle herds and domestic cats, and its persistent infections in them enable species-specific evolution, which might confer human adaptations. However, the unbiased assessment of the adaptations of non-human mammalian H5N1 viruses and their contribution to the human adaptive potential of H5N1 viruses is unclear. In this work, we monitored the evolutionary trajectories of 2.3.4.4b H5N1 in infected non-human mammals to define the mammalian-specific adaptations of this virus and its current potential in establishing human infections.

## RESULTS

**Fig 1:**
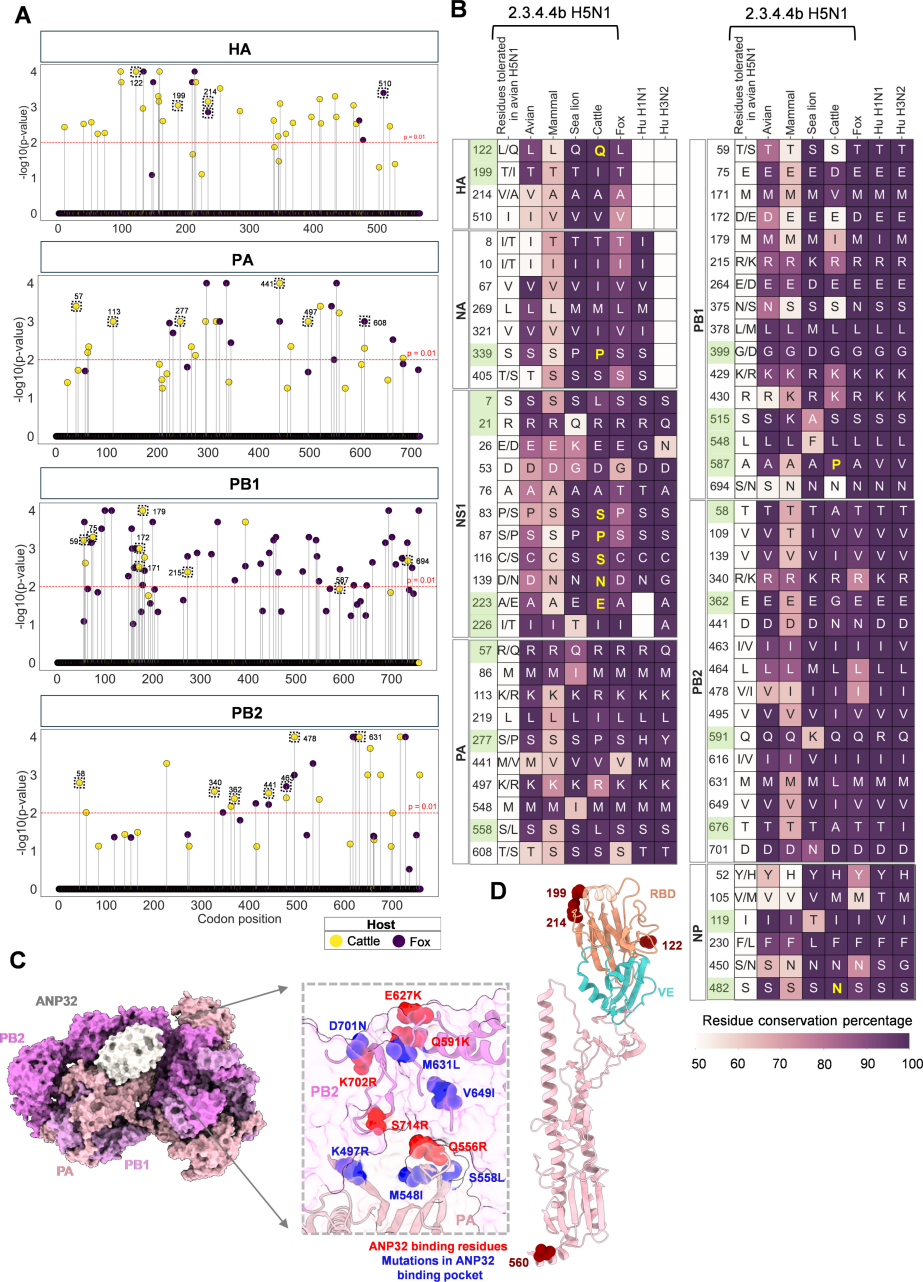
2.3.4.4b H5N1 circulating in non-human mammal species shows rapid evolution and possible adaptive signatures distinct from founder viruses. (A) codon-level evidence of episodic positive selection acting on viral genes of 2.3.4.4b H5N1 isolates from foxes (purple) and cattle (yellow) as inferred by MEME. Each point represents a codon site; the vertical axis shows the statistical significance of episodic selection as –log₁₀(p-value), and the horizontal axis corresponds to codon position. A horizontal red line indicates the significance threshold of *P* = 0.01. Dots above the threshold indicate codons evolving under episodic diversifying selection in at least one foreground branch. Non-synonymous mutations above the threshold are marked in a dashed black box. Plots are shown separately for genome segments of HA, PA, PB1, and PB2. (B) The residue identity and conservation for the positions identified in Panel A are depicted for 2.3.4.4b H5N1 adapted to avian, mammalian, sea lion, cattle, and fox hosts, as well as for the H1N1pdm09 and H3N2 strains adapted to humans. The first column, labeled “residues tolerated in avian H5N1,” notes all amino acid variants at each position in the avian-adapted variant, regardless of coverage. Each cell in the heatmap is annotated with the amino acid residue circulating in at least 50% of the isolates, with the cell color representing the coverage of residue identity. Amino acid substitutions that were likely due to the founder effect have been annotated in yellow in the heatmap. The residue positions representing *de novo* substitutions (residues not tolerated in avian H5N1) are highlighted in green. (C) The surface representation of the ANP32B-bound FluPolA asymmetric dimer is shown, with ANP32B highlighted in white and the remaining subunits annotated (PDB: 8R1J). The zoomed-in inset reveals mutations identified in Fig. 1B located within the ANP32 binding pocket of FluPolA, depicted as blue spheres. Residues directly implicated in ANP32 binding are shown as red spheres. (D) The structure of H5 HA (PDB: 9JMZ) showing the possible nonhuman mammalian adaptations indicated in Panel B.

Upon transmission to non-human mammalian hosts, the avian influenza virus H5N1 accumulates mutations that likely facilitate adaptation to the new cellular environment. To track this process in clade 2.3.4.4b H5N1 viruses, we compared viral genomes and proteins from avian and non-human mammalian hosts (Fig. S1A through C). We first asked which viral genome regions are subject to immediate host-specific selective pressures following spillover. To address this, we performed codon-level selection analyses using the mixed effects model of evolution (MEME) in HyPhy (14, 15), with foreground branches designated based on host metadata (fox or cattle; sea lion-derived sequences were excluded from this analysis due to insufficient sequence data availability). The resulting codon-wise selection profiles revealed marked differences in the extent and distribution of episodic positive selection across the two mammalian hosts (Fig. 1A and Fig. S2). Fox-adapted viruses displayed a broader spectrum of positively selected sites, particularly within the viral polymerase complex (PA, PB1, and PB2) and nucleoprotein (NP), consistent with strong selective pressure for functional adaptation in this host (Fig. 1A). In contrast, cattle-derived viruses showed comparatively fewer selected codons (*P* ≤ 0.01), suggesting either a weaker adaptive response or more recent emergence in the bovine host, with limited time for extensive evolutionary exploration (Fig. 1A). Selection pressures also varied substantially between viral proteins. PB1 and PB2 showed the highest density of positively selected codons across both host types, supporting their well-established roles in host-range restriction and polymerase optimization (Fig. 1A). Several of these sites corresponded to non-synonymous substitutions (highlighted with dashed outlines in Fig. 1A), reinforcing their functional relevance. PA displayed moderate and host-specific selection patterns, while NP and NS1 were more prominently selected in fox-adapted viruses, potentially reflecting differential immune pressure or replication constraints (Fig. 1A and Fig. S2). In contrast, HA and NA exhibited relatively sparse selection in both hosts, implying that antigenic drift or receptor-binding changes may not be the predominant drivers of adaptation during early mammalian spillovers. Collectively, these results highlight the heterogeneity of host-specific evolutionary pressures acting on 2.3.4.4b H5N1 viruses and suggest that the fox represents a more permissive environment for adaptive diversification than cattle.

We further annotated the specific variations (with a minimum of 50% coverage) that appeared once the virus jumped to non-human mammals to determine whether these variations are preadaptive mutations in circulating avian H5N1 or occur *de novo* (the residues different from preadaptive mutations). Due to the sequence sampling constraints, we analyzed fox, cattle, and sea lion viruses individually, and the rest of the non-human mammal viruses have been combined. These residue positions were further compared with avian 2.3.4.4b H5N1, pandemic 2009 H1N1, and seasonal H3N2 viruses. This analysis showed that 2.3.4.4b H5N1 that infected non-human mammals appeared to have multiple *de novo* amino acid substitutions (Fig. 1B). The founder H5N1 viruses determine the successful establishment of infections in the new hosts. To eliminate the possibility of founder effect mutations being propagated in subsequent mammalian infections, we conducted a comprehensive sequence-level mutational mapping after determining evolutionary relationships. We constructed maximum likelihood (ML) phylogeny trees for each H5N1 protein used in this study and mapped the identified mutations onto a heatmap in line with the phylogenetic tree (Fig. S3 and S4). This allowed us to discern adaptations conferred due to amino acid substitutions from founder effects. If a mammalian virus carries a mutation that was already present in closely related avian strains, then the mutation likely did not arise after transmission to the mammalian host. Instead, it was inherited from the avian progenitor, and thus, those mutations were not considered as mammalian (new host) adaptations. Additionally, a mutation found only in a single monophyletic clade of mammalian hosts and not across phylogenetically distinct clusters of the same host likely indicates a founder effect (16). We observed distinct clustering patterns in the phylogeny trees constructed for each protein segment of the 2.3.4.4b H5N1 strain adapted to various host types (Fig. S3 and S4). Specifically, viruses adapted to cattle formed distinct clusters in the phylogenetic tree, whereas sequences from foxes were interspersed among avian viral sequences. This suggested multiple independent transmission events from avian hosts to foxes, in contrast to less frequent transmission events to cattle. The phylogenetic and variant analysis revealed that avian H5N1 sequences that were evolutionarily related to the mammalian viruses did not show the mutations identified in dairy cattle (L122Q [HA], T199I [HA], S558L [PA], T58A [PB2], E362G [PB2], T676A [PB2]), and sea lions (R57Q [PA] and Q591K [PB2]), suggesting the adaptation of the polymerase complex and surface glycoproteins of 2.3.4.4b H5N1 in non-human mammals (Fig. S3A through D). Additionally, several of these mutations are not confined to a single monophyletic clade but are distributed across multiple clades. These observations allowed us to reject the null hypothesis that these mutations do not confer any true adaptation and instead likely represent potential adaptations. Mappings of the mutations that the 2.3.4.4b H5N1 strain acquired upon infecting non-human mammals revealed several substitutions at the functional surfaces of HA and NA, NS1, and polymerase complex proteins (Fig. 1C and D and Fig. S5A and B).

**Fig 2:**
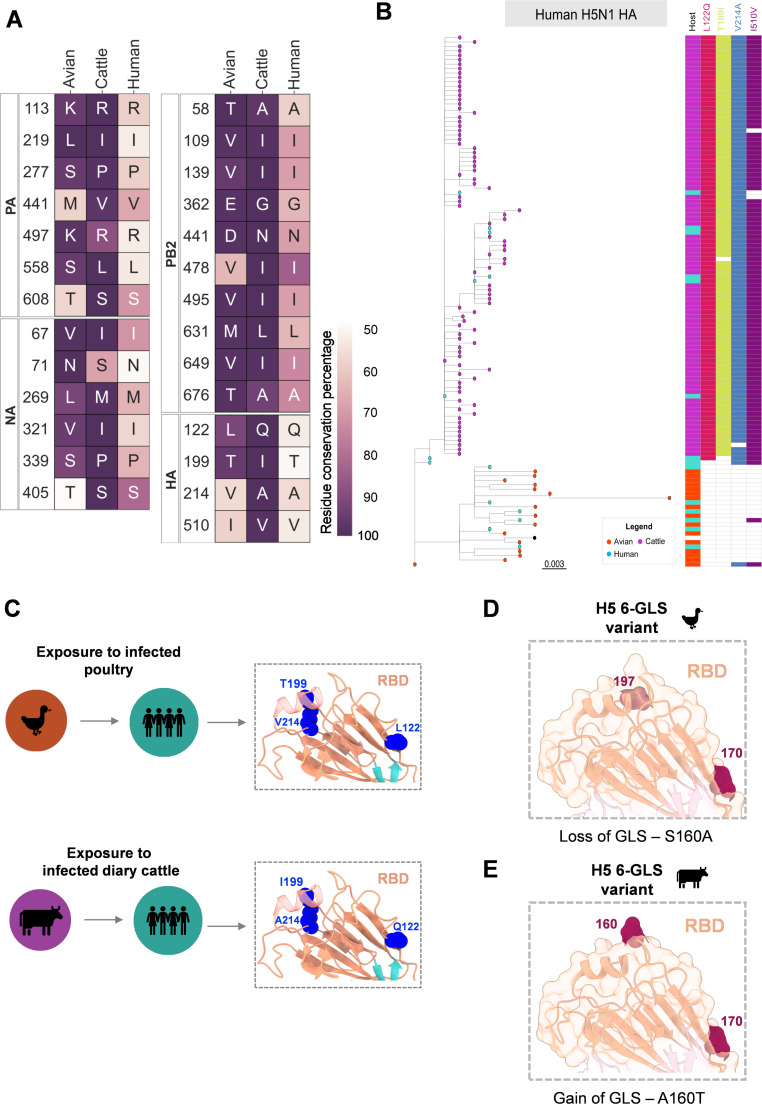
The founder 2.3.4.4b H5N1 strain determines the mutation profile and H5 HA glycan surface plasticity in cattle and human infections. (A) The heatmap illustrates residue conservation at each point of variation in the HA, NA, PA, and PB2 proteins of 2.3.4.4b H5N1 from avian, cattle, and human hosts. Each cell is annotated with the amino acid residue present in at least 50% of the isolates, with the cell color representing the level of residue coverage. (B) ML phylogeny trees constructed for the H5 HA protein using a representative data set of avian (93 sequence clusters with CDHit 99%) and mammalian hosts (cattle: 63; human: 21 clusters with CDHit 100%) infected with 2.3.4.4b H5N1. The nodes of the tree are color-coded by host type. The adjacent heatmap displays the presence of mutations mapped to the corresponding sequences from the phylogenetic tree. (C) The monomeric structure of H5 HA from the trimer assembly (PDB: 3UBE) with the receptor binding domain is shown in orange, the vestigial esterase (VE) domain in cyan, and the rest of the monomer structure in pink. Zoomed-in receptor binding site insets of H5 HA highlight the variations in the RBD of H5 HA when humans are infected with avian-adapted versus cattle-adapted 2.3.4.4b H5N1. GLS – glycosylation sites. (D, E) The glycosylation coverage variants of cattle and avian-adapted 2.3.4.4b H5N1 H5 are mapped onto the RBD of an HA monomer in the H5 trimeric assembly (PDB: 3UBE). The H5 monomer RBD is in a light orange-colored ribbon and surface model. Glycosylation sites (GLS) are shown as red spheres, with the position of the N residue mentioned in H3 numbering. The substitution events leading to the acquisition of these glycosylated variants in cattle and avian hosts are mentioned in the inset below.

As of November 2024, there have been nearly 60 cases of human infections with 2.3.4.4b H5N1 reported to have been either exposed to infected poultry or dairy cattle (12, 13). We analyzed human-infected 2.3.4.4b H5N1 viral sequences and found that these viruses closely resembled the cattle-adapted viral strains, notably in HA, NA, PA, and PB2 (Fig. 2A). To exclude the possibility of founder effect mutations, we constructed an ML phylogeny tree using a non-redundant data set of avian-, cattle-, and human-infected 2.3.4.4b H5N1 H5 HA proteins (Fig. 2B). The human viruses interspersed with both cattle and avian sequences (Fig. 2B). Thus, this pattern suggests at least two possible transmission routes of 2.3.4.4b H5N1 to humans: one avian-mediated and the other through contact with infected dairy cattle. The mutational profile of HA in human-infected 2.3.4.4b H5N1 varied depending on the source of transmission. Human H5N1 found among cattle viral sequences in the phylogenetic tree exhibited all four identified HA mutations (L122Q, T199I, V214A, and I510V) in dairy cattle viruses (Fig. 2B and Fig. S5C). In contrast, human H5N1 sequences distributed among avian viral sequences did not show these mutations. This further indicates that the presence of these four HA mutations, in particular the T199I mutation, in human-infected 2.3.4.4b H5N1 depends on whether the virus originated from a cattle-adapted or avian-adapted H5N1 strain (Fig. 2C).

Intriguingly, in recent cattle infections, A160T mutation appeared in HA (although in low frequency), potentially restoring a glycosylation site at N158 near the sialic acid binding site in the receptor binding domain (RBD) (Fig. 2D and E). Loss of glycosylation at 160 residue position of avian HA in 2.3.4.4b H5N1 confers six glycosylation site variants of H5, which is critical for pairing H5 with long stalk N1 and the functional balance required for virus fitness (9). However, N1 sequences in cattle H5N1 viruses bearing HA with A160T mutation continued to show a long stalk. Also, a recent deep mutational scanning study showed that A160T mutations in 2.3.4.4b H5 HA enable the virus to escape from neutralizing sera (17). This suggests the evolving glycan surface of H5 HA in cattle, which potentially impacts the receptor binding affinity and immune evasion. Importantly, loss of N158 glycosylation site in HA of H5N1 confers increased transmissibility in ferrets and increased binding of H5N1 to the α2,6-linked receptor present in mammalian cells, suggesting that the gain of A160T mutation in cattle viruses could be a potential barrier mutation for human adaptation (18–22).

The influenza A polymerase (FluPolA) also showed significant changes in the functionally important surfaces. The replication platform of influenza consists of an asymmetric dimer of FluPolA heterotrimers, bridged by the host acidic nucleophosphoprotein of 32 KDa (ANP32) (23). Upon binding to host ANP32, the influenza FluPolA switches from transcription to replication. ANP32 appears in A and B isoforms. ANP32A is the only ANP32 family member that efficiently supports FluPolA in birds, while in humans and most other mammalian hosts, isoforms ANP32A and ANP32B support FluPolA activity to varying levels (24). A major barrier to avian influenza virus replication in mammalian cells is the incompatibility of the FluPolA with host-specific ANP32 proteins (25). Structure-based mapping of the non-human mammalian 2.3.4.4b H5N1 mutations of FluPolA (26) showed that two of the residues of the PB2 subunit involved in ANP32 binding were mutated (Fig. S5D and Fig. 1C). These mutations (M631L in cattle and D701N in sea lions) have been previously identified to confer human adaptation of avian influenza viruses. M631L and D701N mutations in the ANP32 binding site allow the FluPolA to utilize both ANP32A and 32B isoforms (24, 27, 28). These mutations are unlike the well-characterized human adaptation E627K, which biases the FluPolA to use ANP32B proteins instead of isoform A (24). Cell line-based studies also showed that D701N mutation facilitates increased adaptation of the avian influenza virus in human cells (24, 29). In addition, a few residues, M548, S558, and K497, in PA and V649 in PB2 of FluPolA, which are in the ANP32 binding pocket (<3.5 Å distance from ANP32 binding surface), were mutated in cattle (S558L and K497R), foxes (V649I), and sea lions (M548I) (Fig. 1C). Analyzing H1N1pdm09 and seasonal human H3N2 FluPolA for these residue positions showed that they tolerated the same amino acids as avian 2.3.4.4b H5N1 (Fig. 1B). Perhaps these PA residues were mutated in cattle viruses for species-specific adaptation in cattle, which might be a barrier to human adaptation.

The innate immune system restricts influenza infection at the acute stages. The NS1 protein of influenza is a crucial host restriction factor to mitigate interferon and other intracellular viral restricting mechanisms (30). NS1 of non-human mammal infected 2.3.4.4b H5N1 acquired notable variations in the RNA-binding domain of NS1. These four mutations (S7L, R21Q, E26K, and D53G) in the RNA-binding domain of the NS1 are *de novo* and host-specific (Fig. S5A and Fig. 1B). This suggests the possible mammalian-specific adaptation of NS1 to facilitate vRNA replication and restrict IFN production. However, these non-human mammalian H5N1 NS1 variations were distinct from human viruses (Fig. 1B) and might be a potential barrier for the dairy cattle viruses to adapt to humans.

**Fig 3:**
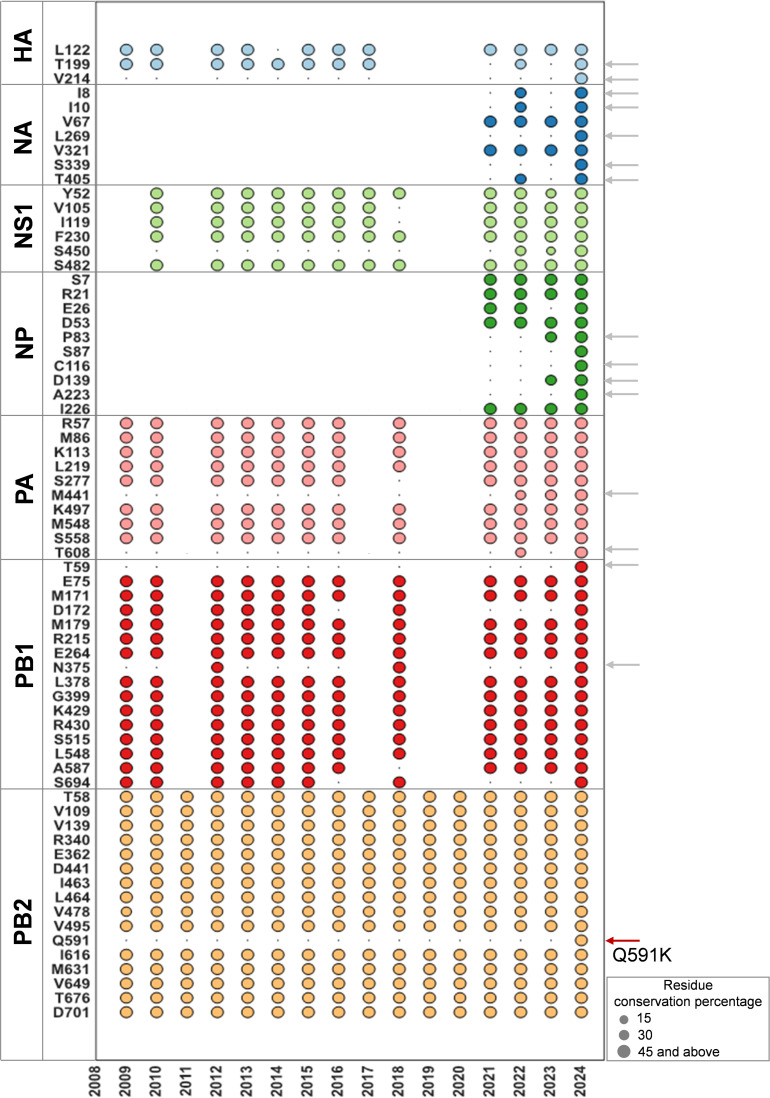
Evolutionary timeline of non-human mammal adaptations in 2.3.4.4b H5N1. The bubble plot displays the variation in the prevalence of non-human mammalian adaptations across different proteins identified in Fig. 1, in mammals infected with previous clades of H5N1. The *x*-axis represents the years sampled, with each bubble corresponding to the number of sequences isolated from mammals infected with H5N1 during that particular year that exhibited the specific adaptation found in mammals infected with the 2.3.4.4b H5N1 clade. Bubbles are color-coded by protein type, and their size indicates the percentage coverage of the mutation. Empty spaces (without bubble representation) represent a lack of high-quality sequence information in that particular year. Arrows on the right side of the plot indicate unique adaptations acquired by the 2.3.4.4b H5N1 clade in non-human mammals. The Q591K in PB2 is a unique adaptation in 2.3.4.4b H5N1, similar to the Q591R human adaptation signature.

**Fig 4:**
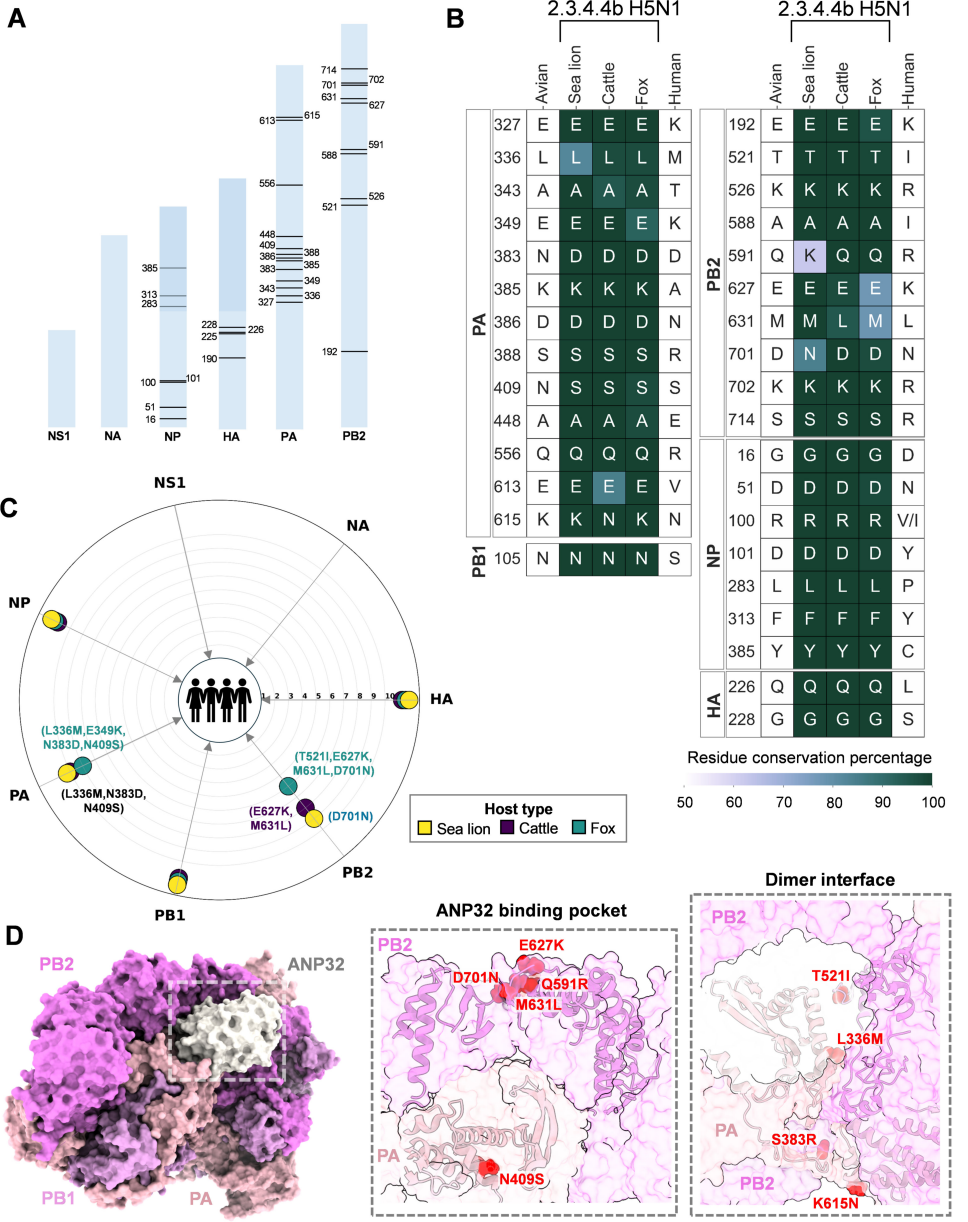
Partial gain of classical human adaptive signatures in PA and PB2 of non-human mammal-adapted 2.3.4.4b H5N1. (A) amino acid positions crucial for human adaptation are mapped onto the length of each protein. The bar lengths reflect the relative lengths of the proteins. For NS1 and NA, specific residues implicated in human adaptation studies were not found and are thus not annotated. (B) Residue conservation maps show the identity and prevalence of residues at positions identified in Panel A in sea lions, cattle, and foxes infected with 2.3.4.4b H5N1. The “human” column indicates the substitutions required at these positions to achieve human adaptation. Each cell is annotated with the residue circulating in at least 50% of the isolates, and cells are color-coded based on the exact coverage value. (C) The radar plot illustrates all the classical human adaptive signatures that have entered circulation, regardless of the coverage of these mutations. The angular axis represents the seven H5N1 proteins investigated in this study, while the radial axis denotes the number of classical human signatures yet to be acquired in each IAV protein. The bubbles are color-coded by host type, and each bubble is annotated with the classical human signatures that have already been acquired by the virus in the corresponding host. (D) Structural mapping of the classical human adaptive signatures already acquired by 2.3.4.4b H5N1 circulating in non-human mammals. The classical human mutations in the PA and PB2 subunits of the FluPolA, identified in Fig. 3C, are mapped onto the ANP32 binding pocket and dimeric interface of PDB:8R1J.

2.3.4.4b H5N1 has transmitted to more diverse mammalian species than previous isolates and has shown successful spread among mammals. We analyzed all the H5N1 sequences that infected non-human mammals across time deposited in GISAID. We found that a majority of the variations were similar between 2.3.4.4b and previous H5N1 clades (Fig. 3). However, H5N1 acquired a few unique variations in non-human mammals with the onset of the emergence of 2.3.4.4b clade H5N1 (Fig. 3). In particular, Q591K in PB2 appeared only in 2.3.4.4b H5N1, which was not seen in previous non-human mammal-infected H5N1 viruses (Fig. 3). The Q591K mutation is similar to the human-adapting mutation in PB2 (Q591R) (24). We then asked whether 2.3.4.4b H5N1 in non-human mammals shows human permissive mutations similar to pandemic 2009 H1N1 and seasonal H3N2 viruses. To understand this, we compared the non-human mammal-adapted mutations of 2.3.4.4b H5N1 (from Fig. 1B) with the H1N1pdm09 and seasonal human H3N2. This analysis revealed that non-human mammal-infected 2.3.4.4b H5N1 signatures (identified in Fig. 1B) converge with human-adapted residue positions of influenza in multiple viral proteins. Several seminal studies, including previous gain-of-function experiments, identified mutations that promote human adaptation of avian HPAI H5N1 (Fig. 4A) (24, 31–52). We further compared whether non-human mammals infected with 2.3.4.4b H5N1 viruses acquired substitutions conferring human adaptation of the virus. The residue conservation map showed that several classical human-adapting mutations have not yet appeared in 2.3.4.4b H5N1 circulating in non-human mammals (Fig. 4B). Intriguingly, N409S in PA, which confers human adaptation by modifying the FluPolA-ANP32 binding site (26, 48), appeared in all circulating viruses in dairy cattle, foxes, and sea lions (Fig. 4B and Fig. 4C). Similarly, highly conserved N383D (cattle, fox, and sea lions) and K615N (cattle) human-adapting mutations were observed in PA (Fig. 4B), which are positioned at the FluPolA dimer interface (Fig. 4D). Also, a few PB2 residues crucial for ANP32 binding showed variations that match human-adapting mutations. The two adapting mutations of PB2 in cattle and sea lions, M631L and D701N (Fig. 4B), enable human adaptation by modifying the ANP32 binding pocket. The sea lion viruses showed acquisition of Q591K (>50% coverage) in PB2 like human-adapting Q591R mutation (Fig. 4B). These adaptations suggest the possible emergence of classical human-adapting mutations in 2.3.4.4b H5N1 of non-human mammals.

Intriguingly, a few specific positions of PA and PB2 proteins of H5N1 in cattle, foxes, and sea lions corresponding to human-adapted positions showed reduced coverage (Fig. 4B), suggesting the existence of additional adaptations that have not yet dominated in circulating viruses. This prompted us to examine less frequent variations in 2.3.4.4b H5N1 of non-human mammals to assess their ability to emerge into novel variants that resemble human-adapted influenza strains. This analysis showed the appearance of additional PA (L336M and E349K in foxes) and PB2 (E627K in foxes and cattle; T521I in foxes) human-adapting variations in circulating non-human mammal viruses (Fig. 4C and D). Considering all these human-adapting mutations in non-human mammal viruses, we found that fox PA and PB2 acquired more human-adapting mutations than cattle and sea lions (Fig. 4C). Cattle viruses tend to show better human adaptation features in PA than PB2 (Fig. 4C). Despite relaxing the residue coverage limit to consider less frequent variations, HA and NP did not possess any adaptations suitable to humans (Fig. 4C).

**Fig 5:**
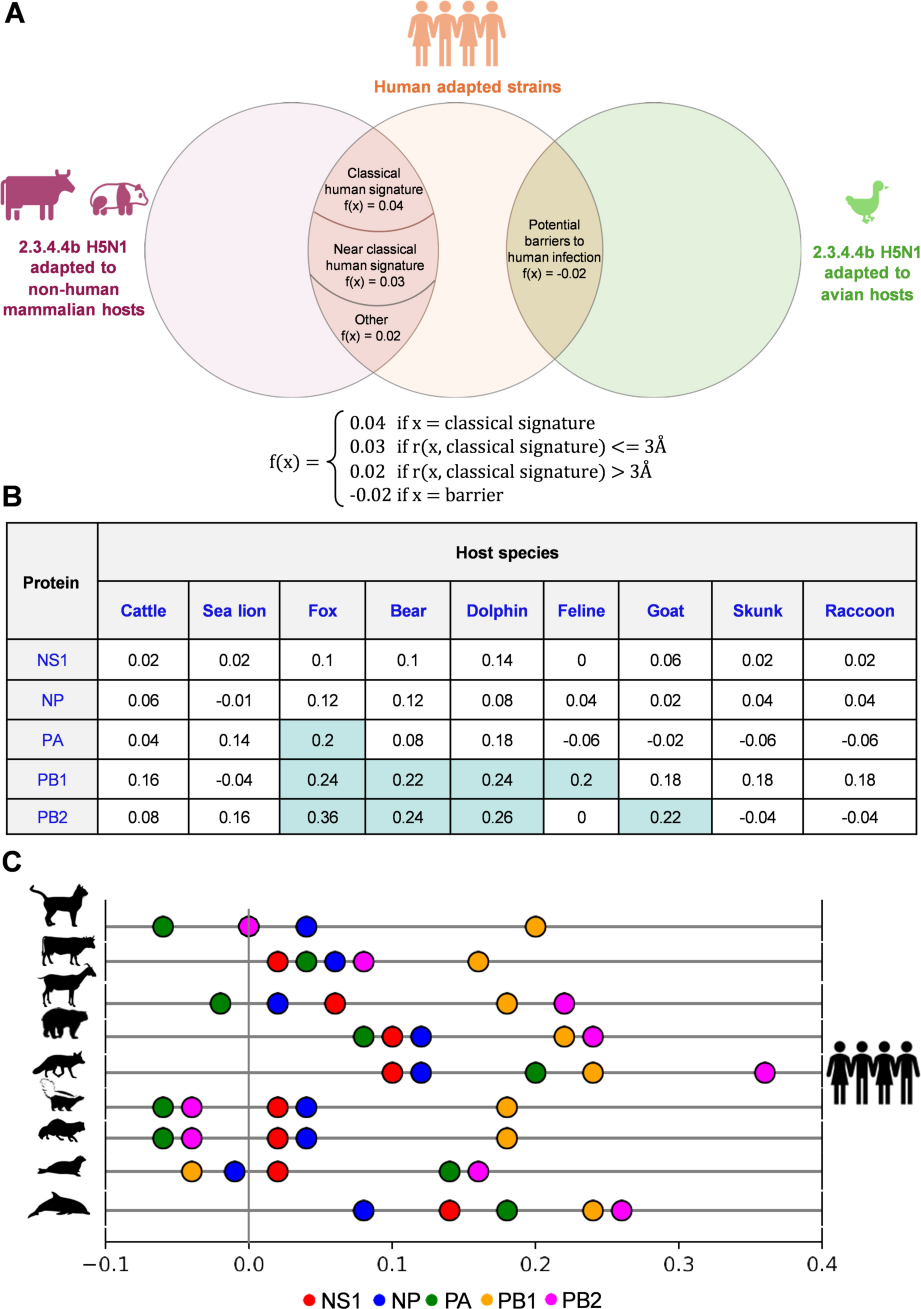
Molecular assessment of human adaptive potential in 2.3.4.4b H5N1 adapted to various non-human mammalian hosts. (A) The Venn diagram shows the classification of adaptations in non-human mammals that mimic human influenza variants, based on their structural similarity to classical human adaptations. The diagram includes weights for each classification, assigned to each mutation x according to the function *f*(*x*). Barrier mutations, which are the mutations that are identical in both human and avian strains but have changed in non-human mammals, are also highlighted in the diagram. (B) The human adaptive potential is calculated using the weights described in Panel A for each adaptation in influenza proteins, with the results displayed across various host types. Cells with a human adaptive potential of 0.2 or higher are highlighted in cyan. (C) The values in Panel B for each protein are plotted on a one-dimensional graph for all host types. Each bubble is color-coded by protein type, with the *x*-axis representing the human adaptive potential. Negative values indicate that the barriers outweigh potential human adaptations, making the variant less potent to adapt to humans compared to the avian variant. A value of zero suggests a balance between barriers and adaptations. A positive value indicates a favorable human adaptive potential, suggesting a higher likelihood of establishing human infection compared to the avian variant.

Furthermore, we evaluated these similarities of non-human mammal 2.3.4.4b H5N1 with human viruses to monitor the human adaptation potential of these viruses. It is reasonable to justify that the greater the number of amino acid similarities of non-human mammal H5N1 viruses with human-adapted viruses, the higher the chance that the virus establishes successful human adaptation. This is based on the assumption that each adaptation that occurred in 2.3.4.4b H5N1 of non-human mammals, which matches human viruses, would have an equal contribution to human adaptation. However, host-specific adaptations of 2.3.4.4b H5N1 in non-human mammals that differ from human adaptations might pose a potential barrier to further human adaptation. We found that some of the residue positions that are identical in avian 2.3.4.4b H5N1 and human H1N1/H3N2 viruses were mutated to adapt for specific non-human mammalian hosts, especially for dairy cattle (such as L219I in PA of dairy cattle, Fig. 1B). We considered these constraints for quantitatively assessing the potential of a non-human mammal H5N1 for gaining human adaptation that likely poses infection risk and spread (Fig. 5A). We considered 2.3.4.4b H5N1 virus sequences from diverse non-human mammals for assessing human adaptive potential. Intriguingly, this analysis showed that fox 2.3.4.4b H5N1 viruses appeared to show greater human adaptive potential in most virus proteins, followed by dolphins, bears, and cattle viruses than all the non-human mammal viruses analyzed (Fig. 5B and C). Also, FluPolA components, PB1, PB2, and PA, of bear, dairy cattle, dolphin, and fox sequences appeared to be acquiring human adaptive potential (Fig. 5B and C). Due to the widespread dairy cattle outbreaks, the circulating 2.3.4.4b H5N1 in cattle might pose a potential human infection risk if these outbreaks continue.

## DISCUSSION

The recent 2.3.4.4b clade H5N1 shows unusual spread in diverse terrestrial and marine mammals and acquired mammal-to-mammal transmission ability. The sporadic human infections due to viruses transmitted from dairy cattle suggest the frequent spillover of this virus to humans. The increasing frequency of these infections raises concerns about potential reassortment events among influenza virus genes, which could lead to the emergence of the next human pandemic influenza A virus (IAV). In this study, we monitored 2.3.4.4b H5N1 adaptive mutations in non-human mammals to define their ability to acquire sustained human-to-human transmission over time. We undertook comprehensive comparisons among the major encoded protein sequences of influenza and hypothesized that host-specific adaptations of 2.3.4.4b H5N1 in non-human mammals might delay successful human infections once they are transmitted. Our observations show the unique evolutionary trajectory of 2.3.4.4b H5N1 in non-human mammals compared to previous viruses. Our study provides key insights into the following critical questions.

Do non-human mammals enable 2.3.4.4b H5N1 to attain substitutions favoring human adaptations? Several non-human mammal species acquired classical human-adapting mutations, similar to the 2009 human pandemic H1N1 and H3N2. In particular, PA and PB2 of non-human mammal viruses acquired several classical human-adapting mutations (Fig. S6). These mutations in PA and PB2 expand their ability to use both isoforms of ANP32, and the 2.3.4.4b H5N1 is no longer restricted to host types with dominant ANP32B expression. This could explain why we see atypical hosts for 2.3.4.4b H5N1. This is a major concern as the range of non-human mammals that could serve as intermediate hosts for influenza viruses is expanding. We anticipate that the diverse non-human mammal host range and the ability of mammal-to-mammal transmission of 2.3.4.4b H5N1 could enable the evolution of more human adaptive mutations.

What is the selection pressure on specific proteins of 2.3.4.4b H5N1 in non-human mammals? We found several residue positions in PA and NS1, which acquired species-specific adaptations that do not match human adaptations. These residues are identical in avian and human viruses but differ in non-human mammals. Perhaps PA and NS1 species-specific adaptations could be one of the factors contributing to the mild symptoms in humans who were infected with viruses circulating in dairy cattle. Interestingly, H5 HA shows the least adaptations in non-human mammals but rapidly changes the glycan surface near the receptor binding site. The HA glycosylation pattern and the NA stalk length likely drove the evolution of 2.3.4.4b clade H5N1, which differs from previous H5N1 isolates. The glycosylation sites involving 158 (158–160) and 197 (197–199) residues of HA have been evolving in gs/GD lineage H5 viruses and determine the major H5 variant types circulating at a given time. The dynamics of HA surface glycan patterns need to be monitored and examined if these changes bring a sudden shift in HA’s receptor binding specificity or ability to bind diverse receptors on human cells.

Which non-human mammal H5N1 is closely related to human influenza viruses and likely shows greater infection risk? This study assessed the human adaptive potential of several non-human mammals infected with 2.3.4.4b H5N1 by considering the variations in most genome segments. Interestingly, 2.3.4.4b H5N1 in bears, dolphins, and foxes shows better human adaptive potential than other non-human mammals. PA, PB1, and PB2 rapidly gain human adaptations in H5N1 of non-human mammals. In particular, the adaptations of PB2 of H5N1 from foxes, dolphins, and bears show greater human adaptive potential (Fig. S6). Dairy cattle viruses are also evolving human adaptations in polymerase complex proteins.

### Study limitations

In this study, we focused exclusively on the molecular aspects of human adaptive potential. Assuming that the likelihood of exposure to non-human mammals is constant, we evaluated the potential of 2.3.4.4b H5N1 that adapted to non-human mammals to establish infection in humans. Our findings suggest that viruses adapted to foxes and dolphins exhibit significantly greater potential for human adaptation compared to those adapted to cattle, goats, and raccoons. However, it is crucial to consider that the probability of human exposure to these wild animals is much lower than that of domesticated animals like dairy cattle and goats. While assessing adaptive potential and barriers, we recognize that mutations categorized as “barriers” may not necessarily prevent human infection, as they could rapidly mutate into human-permissive variants post-infection. The ability to fully understand these mutations is limited by restrictions on gain-of-function experiments. Also, our observations of rapid glycan surface evolution of 2.3.4.4b H5 HA need functional validation to establish the significance of these changes on receptor specificities. Nonetheless, given the evolving host range of the 2.3.4.4b H5N1 strain, monitoring even distant possibilities of human exposure remains critical.

## MATERIALS AND METHODS

### 2.3.4.4b H5N1 and human influenza data sets

A reproducible pipeline was developed to streamline the data analysis and is accessible via GitHub. Protein sequence data sets for HA, NA, NS1, NP, PA, PB1, and PB2 from 2.3.4.4b H5N1, sampled up until August 1, 2024, were obtained from the EpiFlu database on GISAID.org for both avian and non-human mammalian hosts (sea lions, dairy cattle, and foxes) (53). These three host species were selected due to the constraints on sequence availability and due to their unusual roles as hosts for IAVs, their diverse ecological niches, and the sustained transmission observed among these mammals. Notably, since April 2024, the Centers for Disease Control and Prevention and the US Department of Agriculture (USDA) have reported instances of clade 2.3.4.4b H5N1 infection in dairy cattle, and these newly identified sequences were included in our analysis. To assess the human adaptive potential of the 2.3.4.4b H5N1 variants circulating in these non-human mammalian hosts, we performed a comparative analysis using sequences of H1N1 (pdm09H1N1) and seasonal H3N2, the predominant human influenza strains known for sustained human-to-human transmission. This comparative data set was also sourced from the EpiFlu database on GISAID.org. To ensure a comprehensive global analysis, no geographical restrictions were applied during data set acquisition. Focusing on residue-level variability in influenza virus proteins across different host types, we retained and analyzed only full-length, high-quality sequence data (excluded proteins like protein). In total, 7,000 sequences of 2.3.4.4b H5N1 from avian species, 820 sequences from non-human mammals (distributed among sea lions, cattle, and foxes), and 35000 sequences of human H1N1 and H3N2 were analyzed in this study.

### Protein residue conservation maps of H5N1 and human influenza viruses

To generate the residue conservation maps in Fig. 1B, amino acid variations between avian and mammalian-adapted 2.3.4.4b H5N1 proteins were identified by aligning the consensus sequences of each host type. Multiple sequence alignment (MSA) was performed using Clustal Omega, and consensus sequences were generated using the EMBOSS Cons package. After identifying positions under selection pressure in non-human mammalian hosts, we assessed the extent of adaptation by examining each sequence in the data set. If a particular residue was present in at least 50% of sequences at a given position, it was used to annotate the corresponding cells in the heatmap. Coverage was expressed as a percentage to normalize differences in data set size across host types and proteins. The intensity of each heatmap cell reflected the exact percentage of residue coverage. This analysis included all avian, non-human mammalian, and human (pdm09H1N1 and H3N2) sequences that met quality criteria. The residue conservation maps were visualized as heatmaps using the Seaborn module in Python.

For the residue conservation map in Fig. 3B, residue positions crucial for human adaptation were identified through a literature review. These positions were then analyzed for each host type, with coverage determined using the same methodology as in Fig. 1B. The structures were analyzed using ChimeraX. The HA residue numbering was adapted from the Dadonaite et al. study (17).

### Lineage-specific selection analysis using mixed effects codon models

To identify codons undergoing episodic positive selection in the genes of clade 2.3.4.4b H5N1 viruses, we employed the MEME implemented in the HyPhy package (v2.5.63) (14, 15). Nucleotide alignments for each gene were generated using MAFFT v7.505 with codon-aware alignment and manually inspected to ensure correct reading frames and codon alignment. Corresponding ML phylogenetic trees were inferred using IQ-TREE2 (v2.2.6) under the GTR + G substitution model with 1,000 ultrafast bootstrap replicates (54). Host-specific episodic selection was tested by designating internal branches corresponding to either fox-derived or cattle-derived isolates as the foreground, with all other branches treated as the background. MEME detects sites evolving under positive selection on a subset of lineages by fitting a mixed-effects codon model that allows variation in dN/dS ratios both across sites and lineages (14). Sites with *P* ≤ 0.01 (LRT) were considered to be under statistically significant episodic positive selection. For each gene, codons under selection were plotted as lollipop plots using the matplotlib and seaborn modules of Python.

### Phylogenetic trees, mutational patterns, and founder effect

Sequences for the seven 2.3.4.4b H5N1 proteins (HA, NA, NS1, NP, PA, PB1, and PB2) were downloaded from GISAID, categorized by host species. Partial sequences and those with non-standard amino acids were removed as part of quality control. A non-redundant representative data set was generated by clustering sequences using CD-Hit, enforcing a sequence identity cut-off of 99% for avian hosts and 100% for mammalian hosts (55). MSA was performed using Clustal Omega. The alignments were processed using IQ-TREE2 to construct ML phylogenetic trees. The software automatically determined the most suitable substitution model, ensuring accurate modeling of evolutionary relationships. To evaluate the statistical support for each branch in the resulting tree, 1,000 ultrafast bootstrap replicates were conducted, providing robust confidence estimates for the inferred phylogeny. The comprehensive results, including the ML tree and bootstrap values, were systematically saved with appropriate prefixes for subsequent analysis and interpretation. To provide a comprehensive overview of the phylogenetic relationships and associated host information, the phylogenetic tree was visualized using the ggtree package in R (56). The geom_tree function was used to plot the tree, and a scale bar was added using geom_treescale. Metadata associated with the phylogeny tips, including host and mutation information, was generated using custom scripts in Python and then used to annotate the tree. The gheatmap function was used to add a heatmap to the phylogenetic tree, incorporating the metadata columns. The heatmap was customized to align with the tree layout and display relevant metadata, enhancing the visualization of phylogenetic relationships and associated host information. By following these methods, we effectively constructed and visualized the phylogenetic tree, integrating key metadata to provide a detailed overview of the evolutionary relationships and host information.

### Identifying non-human mammalian adaptations unique to 2.3.4.4b H5N1

The evolutionary timeline for the adaptations depicted in Fig. 2 was constructed to explore whether the adaptations observed in non-human mammals infected with the 2.3.4.4b H5N1 strain are unique to this clade or represent common attributes of H5N1 infections across different clades. For this analysis, we downloaded H5N1 genomes from non-human mammalian hosts without imposing any clade restrictions. After applying quality control measures to the data set, we obtained sequence coverage starting from 2009. For each protein, we assessed the percentage of sequences in the data set for a given year that exhibited the adaptations uncovered in Fig. 1. The coverage of these mutations was plotted year-wise as a bubble plot, with the bubble radii representing the percentage coverage for the seven proteins investigated in this study from 2009 to 2024. The bubble plot was created using the Seaborn module in Python.

### Assessment of the human adaptive potential of 2.3.4.4b H5N1

A simple approach to assess human adaptive potential might involve subtracting the number of barriers from the total number of adaptations, assuming that each adaptation contributes equally to human adaptation. However, numerous studies highlight that acquiring a single classical signature, such as the E627K mutation in PB2, can significantly enhance IAV fitness in human cells. Therefore, we aimed to establish weights for each type of adaptation and barrier. To compare the molecular adaptive potential of 2.3.4.4b H5N1 strains circulating in non-human mammals with that in humans, we weighted the adaptations based on their classification as classical signatures, their proximity to these signatures, and their potential to act as barriers.

Categorizing the adaptations as:

Number of classical signatures = *x*

Number of adaptations within 3 Å of classical signatures = *y*

Number of adaptations beyond 3 Å of classical signatures = *z*

Number of potential barriers = *l*

The mathematical expression ax + by + cz − dl where a, b, c, and d belong to the interval (0.1), was evaluated across various scenarios, including cases where barriers outnumbered adaptations (resulting in ax + by + cz − dl <0) and instances where adaptations outweighed barriers (resulting in ax + by + cz − dl > 0). The weights for these mutations were empirically determined to be: a = 0.04, b = 0.03, c = 0.02, and d = 0.02. Custom scripts were developed in Python to automate the process of assigning weights to each mutation within each protein across different host types.

## Data Availability

All data generated and analysed in this study are included in the main and supplementary figures of this manuscript. All genome sequences and associated metadata in this dataset are published in GISAID’s EpiFlu database. GISAID EPI_SET identifier information is provided below. GISAID Identifier: EPI_SET_250629ep; Data Snapshot: EPI_SET_250629ep is composed of 7,661 individual viruses. The collection dates range from 14 April 2011 to 28 March 2024; data were collected in 75 countries and territories. GISAID Identifier: EPI_SET_250629bd; Data Snapshot: EPI_SET_250629bd is composed of 219 individual viruses. The collection dates range from 1 September 2021 to 1 April 2024; data were collected in 18 countries and territories. GISAID Identifier: EPI_SET_250629bh; Data Snapshot: EPI_SET_250629bh is composed of 221 individual viruses. The collection dates range from 17 May 2021 to 1 January 2024; data were collected in 15 countries and territories. GISAID Identifier: EPI_SET_250629df; Data Snapshot: EPI_SET_250629df is composed of 15 individual viruses. The collection dates range from 23 January 2023 to 26 August 2023; data were collected in three countries and territories.

## SUPPLEMENTAL MATERIAL

Supplemental figures spectrum.00948-25-s0001.pdf Figures S1 to S6.

Table S1 spectrum.00948-25-s0002.xlsx Sources of avian data sets.

Table S2 spectrum.00948-25-s0003.xlsx Sources of fox data sets.

Table S3 spectrum.00948-25-s0004.xlsx Sources of mammalian data sets.

Table S4 spectrum.00948-25-s0005.xlsx Sources of sea lion data sets.

ASM does not own the copyrights to Supplemental Material that may be linked to, or accessed through, an article. The authors have granted ASM a non-exclusive, world-wide license to publish the Supplemental Material files. Please contact the corresponding author directly for reuse.
