# Supplementary material for: Decoding non-human mammalian adaptive signatures of 2.3.4.4b H5N1 to assess its human adaptive potential
Source: Microbiol Spectr. 2025 Aug 11;13(9):e00948-25. doi: 10.1128/spectrum.00948-25 (PMC12403652; doi:10.1128/spectrum.00948-25)
Supplement: Supplemental figures — Figures S1 to S6. [file spectrum.00948-25-s0001.pdf]

SUPPLEMENTAL FIGURES

A

	GISAID	GISAID	GISAID	NCBI GISAID	GISAID
**Protein**	**Avian**	**Mammals**	**Sea lion**	**Cattle**	**Fox**
HA	7941	669	16	372	168
NA	7192	630	15	386	150
M	29	-	0	321	45
PB1	7274	625	14	366	190
PB2	7087	638	15	379	190
PA	7487	634	15	362	197
NP	7777	692	16	323	132
NS1	7670	691	16	335	120

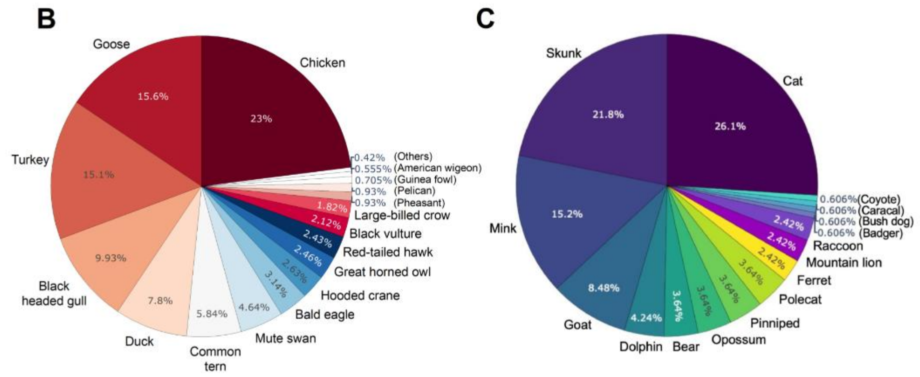

Figure S1: Sequence dataset information considered for analyzing the 2.3.4.4b H5N1 sequences from avian and non-human mammalian species. A. Table presents the number of high-quality, full-length sequences for the five 2.3.4.4b H5N1 proteins investigated in this study. The sequences are categorized by isolates from avian, mammalian, and other hosts, and sourced from GISAID.org. **B**. The species composition within the avian host category is illustrated by a pie chart. Each sector is color-coded and labeled by species, with annotations indicating the percentage of sequences from each species. **C**. Pie chart depicting the species composition of mammalian hosts (except cattle, foxes, and sea lions) of 2.3.4.4b H5N1 is constructed as shown in Fig S1B.

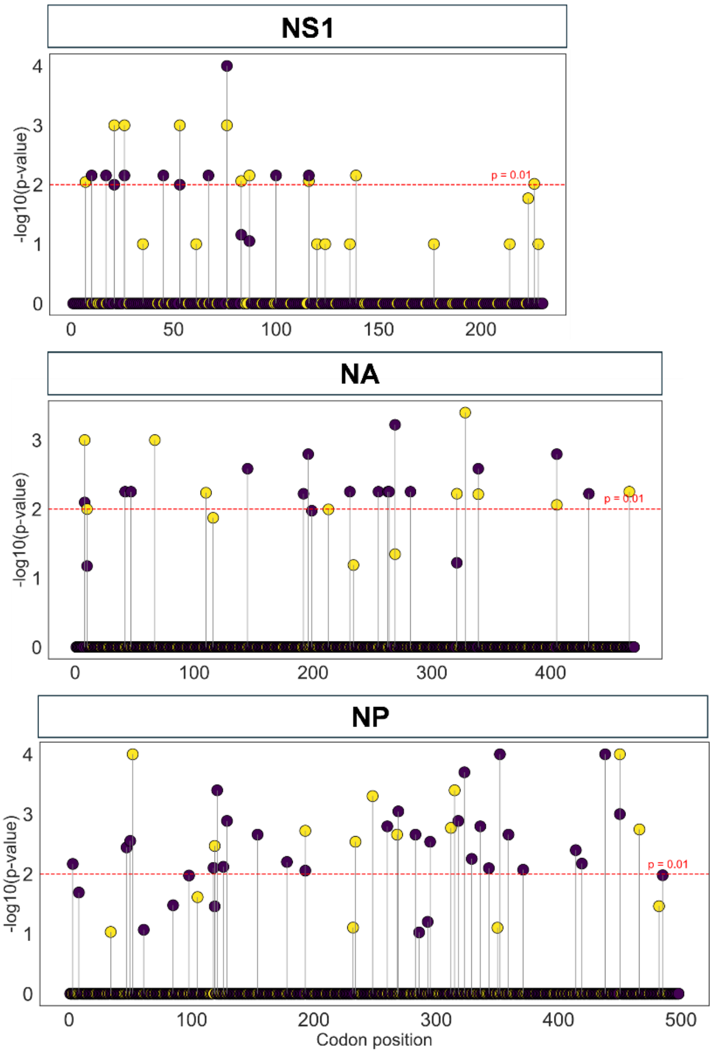

Figure S2: Codon-level mutational analysis and positive selection of NS1, NA, and NP of 2.3.4.4b H5N1 in cattle and foxes. Codon-level evidence of episodic positive selection acting on viral genes of 2.3.4.4b H5N1 isolates from foxes (purple) and cattle (yellow) as inferred by MEME. Each point represents a codon site; the vertical axis shows the statistical significance of episodic selection as $-\log_{10}$(p-value), and the horizontal axis corresponds to codon position. A horizontal dashed red line marks the significance threshold of $p = 0.01$. Dots above the threshold indicate codons evolving under episodic diversifying selection in at least one foreground branch. Sites outlined with a dashed black box represent non-synonymous mutations. Plots are shown separately for NA, NS1, and NP.

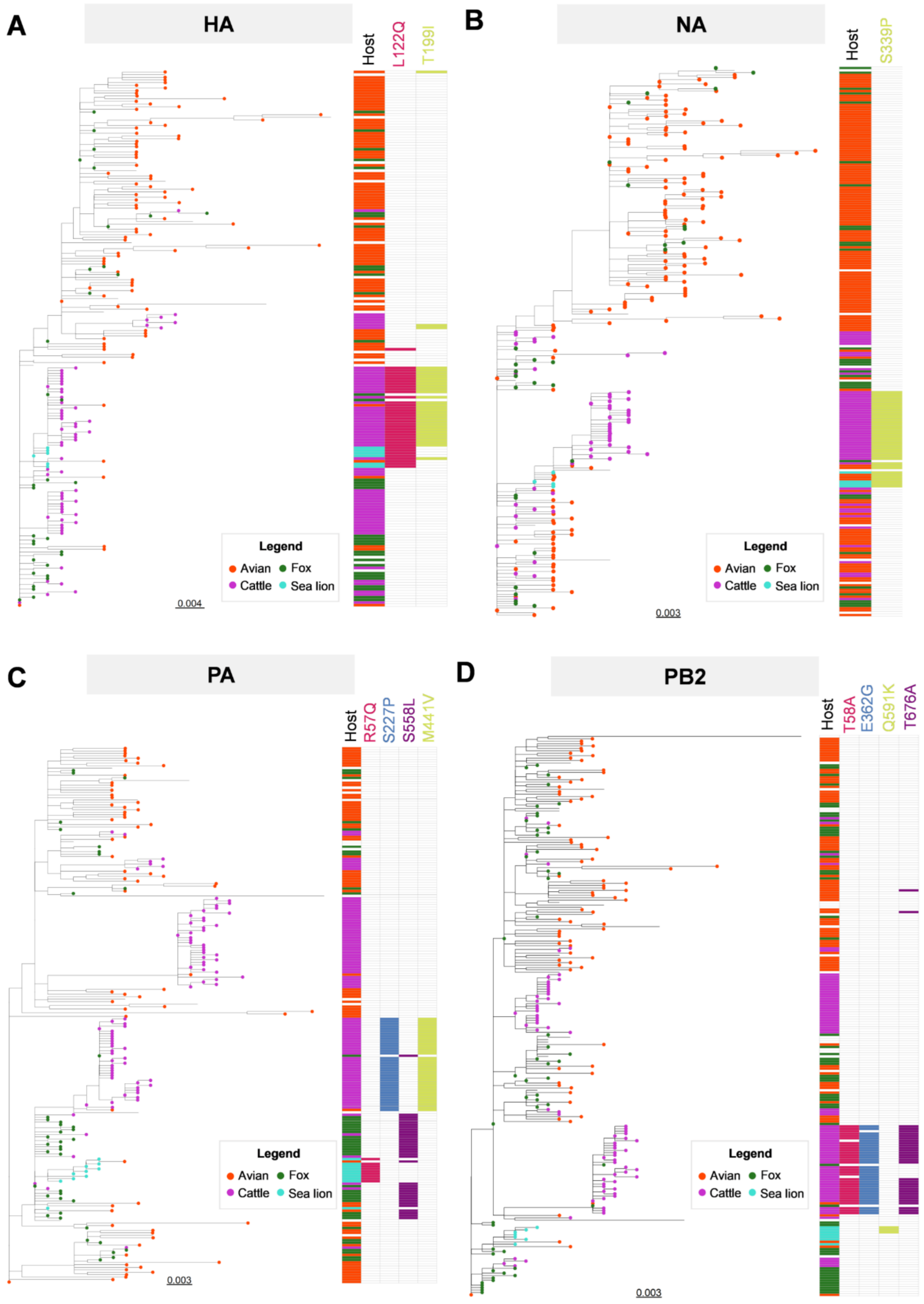

Fig S3. Phylogenetic and founder effect analysis of 2.3.4.4b H5N1 proteins – HA, NA, PA, and PB2. A. Maximum likelihood phylogeny trees constructed for the H5 HA

protein using a representative dataset of avian (97 sequence clusters with CDHit 99%) and mammalian hosts (Cattle: 61, Fox: 80, Sea lion: 14 clusters with CDHit 100%) infected with 2.3.4.4b H5N1. The nodes of the tree are color-coded by host type. The adjacent heatmap displays the presence of mutations (highlighted in green in Fig. 1B) mapped to the corresponding sequences from the tree. **B, C, D** Maximum likelihood phylogeny trees and corresponding heatmaps for the NA, PA, and PB2 proteins, generated as described for the H5 HA protein. NA dataset composition (Avian – 323 clusters with CDHit 99%, Cattle – 57, Fox – 36, Sea lion - 4 clusters with CDHit 100%) ; PA dataset composition (Avian – 79 clusters with CDHit 99%, Cattle – 85, Fox – 92, Sea lion - 20 clusters with CDHit 100%) ; PB2 dataset composition (Avian – 96 clusters with CDHit 99%, Cattle – 73, Fox – 124, Sea lion – 7 clusters with CDHit 100%).

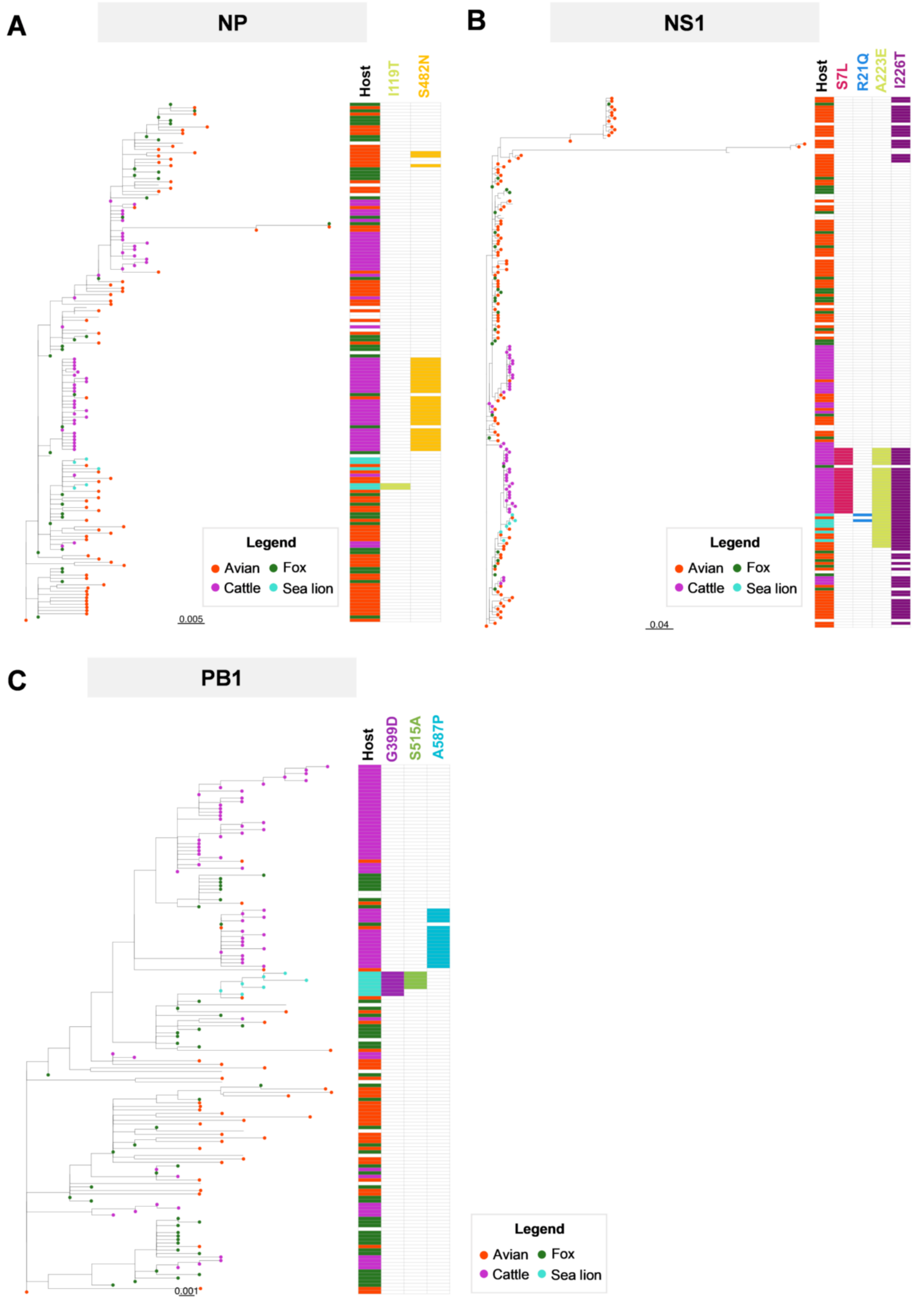

Fig S4. Evolutionary and founder effect analysis of 2.3.4.4b H5N1 proteins – NP, NS1, and PB1. A. Maximum likelihood phylogeny trees constructed for the NP protein

using a representative dataset of avian (70 sequence clusters with CDHit 99%) and mammalian hosts (Cattle: 49, Fox: 40, Sea lion: 5 clusters with CDHit 100%) infected with 2.3.4.4b H5N1. The nodes of the tree are color-coded by host type. The adjacent heatmap displays the presence of mutations (from Figure 1B) mapped to the corresponding sequences from the tree. **B, C.** Maximum likelihood phylogeny trees and corresponding heatmaps for the NS1 and PB1 proteins, generated as described for the NP HA protein. NS1 dataset composition (Avian – 418 clusters with CDHit 99%, Cattle – 55, Fox – 33, Sea lion - 8 clusters with CDHit 100%); PB1 dataset composition (Avian – 47 clusters with CDHit 99%, Cattle – 58, Fox – 46, Sea lion – 7 clusters with CDHit 100%).

A

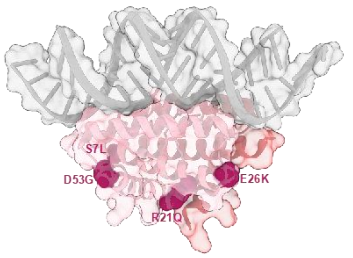

B

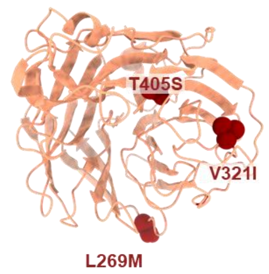

C

HA position	Host type	Residues tolerated	Background frequency	Observed frequency	p-value
122	Sea lion	Q	-	-	-
	Cattle	(Q, L)	(0.5, 0.5)	(0.599, 0.401)	5.26E-01
	Fox	(Q, L)	(0.5, 0.5)	(0.951, 0.0484)	6.16E-01
199	Sea lion	T	-	-	-
	Cattle	(I, T)	(0.5, 0.5)	(0.606, 0.393)	5.26E-01
	Fox	(T, I)	(0.5, 0.5)	(0.951, 0.0484)	6.16E-01
214	Sea lion	A	-	-	-
	Cattle	(A, T, V)	(0.33, 0.33, 0.33)	(0.637, 0.331, 0.031)	5.27E-01
	Fox	(A, V)	(0.5, 0.5)	(0.825, 0.174)	5.63E-01
510	Sea lion	V	-	-	-
	Cattle	(V, I)	(0.5, 0.5)	(0.937, 0.06)	5.62E-01
	Fox	(V, I)	(0.5, 0.5)	(0.728, 0.271)	5.51E-01

D

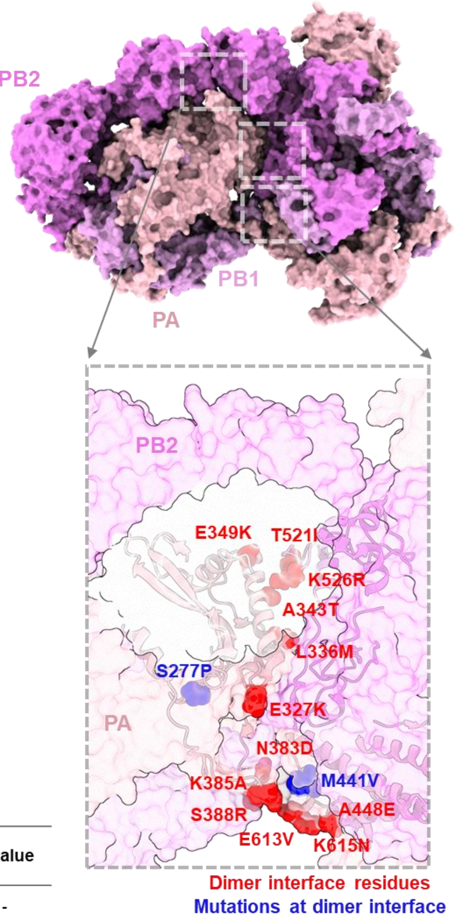

Figure S5: Structural mapping of non-human mammal adaptations of 2.3.4.4b H5N1. **A.** The surface representation of NS1 dimer bound to dsRNA (in grey) (PDB: 2ZKO) is shown. The non-human mammalian adaptations in NS1, identified in Figure 1, are marked as magenta spheres on one of the monomers. **B.** The surface representation of the N1 tetrameric head is shown (PDB:2HTY), with one monomer

highlighted in orange. Zoomed-in insets show the receptor-binding domain of the N1 monomer, along with the mutations as red spheres in this domain. The positions are annotated to highlight the mutation in non-human mammals from the avian variant. **C**. Residue preference at select positions in the HA protein across host species (sea lion, cattle, and fox). For each HA position, the amino acid(s) tolerated in each host are listed alongside the background frequency (assumed uniform under neutral evolution) and the observed frequency in the dataset. A binomial test was used to determine whether the observed frequency of specific residues significantly deviated from expectations under the null hypothesis of neutral evolution. For positions with multiple tolerated residues, the expected background frequency was calculated assuming equal probabilities. **D**. The surface representation of the FluPolA asymmetric dimer is shown, with the subunits—PA, PB1, and PB2—annotated (PDB: 8R1J, chain G of ANP32B hidden). The zoomed-in inset highlights the mutations from Fig. 1B that occur at the dimeric interface (blue), and the residues required for the interface contacts (red).

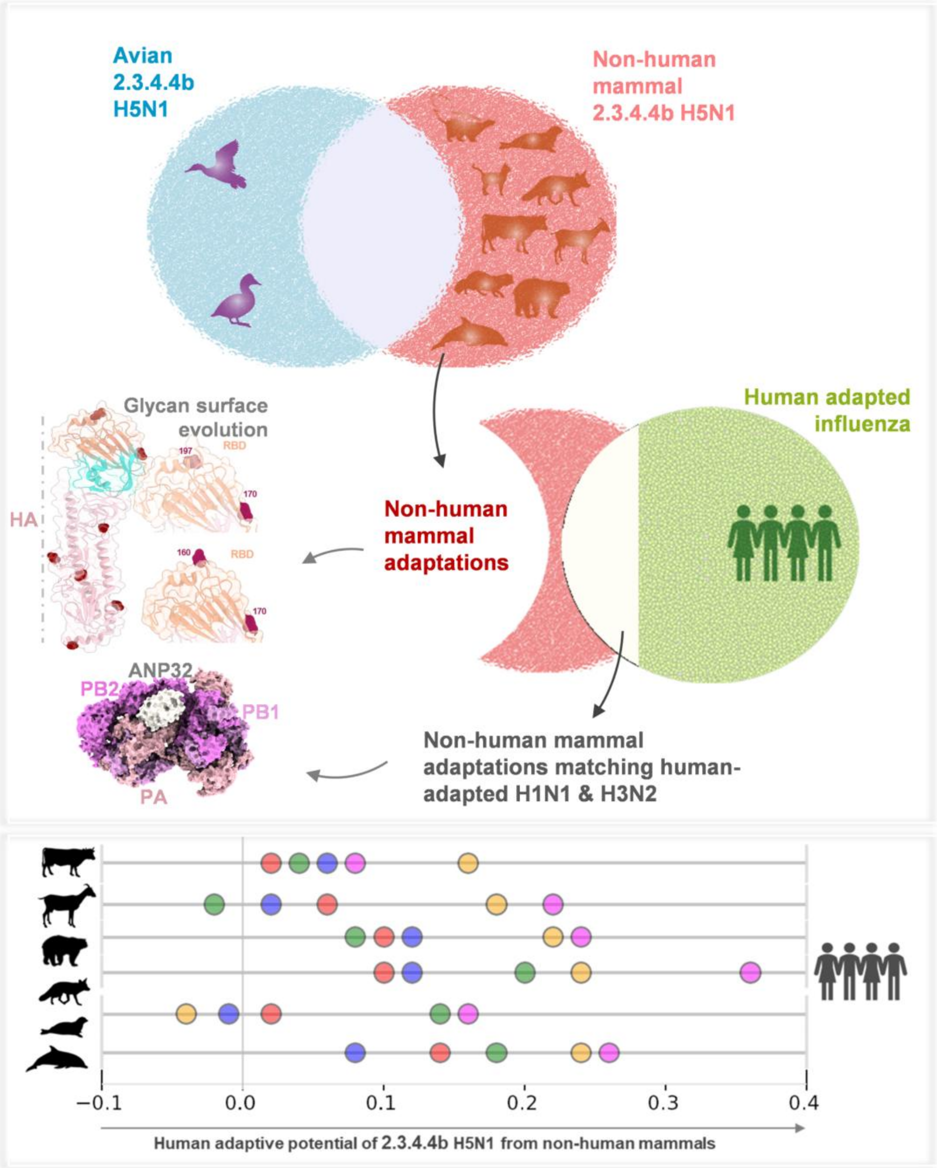

Figure S6: Emergence of possible human adaptations in 2.3.4.4b H5N1 circulating in non-human mammals. Cartoon representation showing the emergence of mammalian adaptations in 2.3.4.4b H5N1 after transmission from avian species and how these mammalian adaptations might enable human adaptations in 2.3.4.4b H5N1. The color bead chart represents the relative human adaptation potential of influenza proteins from 2.3.4.4b infected non-human mammalian species.
